# Anticancer Activity and Mechanism of Action of *Couroupita guianensis* Bark Decoction in Gastric Adenocarcinoma Cancer Cell Line

**DOI:** 10.3390/ijms25179183

**Published:** 2024-08-24

**Authors:** Simona Pisanti, Serena Penna, Silvia Sposito, Tiziana Esposito, Teresa Mencherini, Rita Celano, Tania Re, Rita Patrizia Aquino, Rosanna Martinelli

**Affiliations:** 1 Department of Medicine, Surgery and Dentistry ‘Scuola Medica Salernitana’, University of Salerno, 84081 Baronissi, SA, Italy; spisanti@unisa.it (S.P.); spenna@unisa.it (S.P.); ssposito@unisa.it (S.S.); 2 Department of Pharmacy, University of Salerno, 84084 Fisciano, SA, Italy; tesposito@unisa.it (T.E.); tmencherini@unisa.it (T.M.); rcelano@unisa.it (R.C.); aquinorp@unisa.it (R.P.A.); 3 UNESCO Chair Salerno Plantae Medicinales Mediterraneae, University of Salerno, 84084 Fisciano, SA, Italy; tania.re77@gmail.com; 4 UNESCO Chair “Health Anthropology, Biosphere and Healing Systems”, University of Genova, 16126 Genova, GE, Italy

**Keywords:** *Couroupita guianensis*, apoptosis, cell cycle, cytotoxicity, autophagy, AGS cells, gastric cancer, traditional medicine

## Abstract

*Couroupita guianensis*, a medicinal plant autochthonal to South America and South India, is widely used in the ethnomedicine of the indigenous peoples of these regions thanks to its alleged antimicrobial, anti-inflammatory, antioxidant and wound-healing properties. The majority of studies have mainly analyzed organic extracts of the Indian plant’s flowers and leaves, with limited research on its bark decoction, traditionally used in Amazonian shamanic medicine. In this study, we investigated the anticancer effects of the bark decoction and its main fractions obtained through chromatographic separation, as well as the underlying molecular mechanisms in AGS gastric cancer cells. Viability, cell proliferation, cell cycle, apoptosis and protein expression related to these processes were evaluated. Both the bark decoction and fraction III significantly inhibited cell viability, and the cytotoxic effect was linked to cell cycle blockade and the induction of apoptosis also through an engulfment of the autophagic flux. Increased expression or activation of the key proteins (p53, p21, cdk2, Bak, caspases, pAMPK, pAkt, beclin, p62 and LC3BII) involved in these processes was observed. The results obtained confirmed an important anticancer effect of *C. guianensis* bark decoction, providing scientific validation for its use in traditional medicine and highlighting its potential as a therapeutic agent against gastric cancer.

## 1. Introduction

*Couroupita guianensis* Aubl., commonly known as the cannonball tree, has attracted considerable attention due to its potential medicinal applications. The plant is a member of the Lecythidaceae family and is indigenous to the rainforests of Central and South America, South India, and Malaysia [1]. It is cultivated extensively in tropical regions across the globe. The tree is esteemed for its extensive range of therapeutic benefits. *C. guianensis* is employed in traditional shamanic Amazonian medicine with the name of ‘ayauma’ for the treatment of a range of ailments, including infections (skin and upper airways, malaria, tuberculosis), gastric issues (gastritis, diarrhea, meteorism), inflammatory conditions, pain (headache, back pain) and skin diseases, among the others. It is also used in Ayurvedic medicine to treat the so-called ‘cold’ ailments [2,3,4]. This is achieved using various parts of the tree, namely flowers, leaves, bark and roots. Despite its disagreeable odor, the fruit pulp is employed in the treatment of colds, wounds, headaches and stomach aches and exhibits antibacterial and antifungal properties [5,6]. Organic extracts from different parts of the plant, including leaves, flowers and fruits, show significant anti-inflammatory and antioxidant properties thanks to a variety of bioactive phytochemicals [7].

Beyond its intrinsic biological properties, *C. guianensis*, thanks to its high content of flavonoids, phenolic groups and terpenoids, has been effectively used as a reducing and capping green agent to synthesize silver and other metal nanoparticles that effectively inhibit growth and biofilm formation in human pathogenic bacteria and have antioxidant and anticancer activities in vitro [8,9,10,11].

We recently reported the complete phytochemical profile of a *C. guianensis* aqueous bark decoction for the first time, as used in the shamanic traditional Amazonian medicine. A new ellagic acid derivative, namely 4-(2″-O-sulfate- β-D-glucuronopyranosyl) ellagic acid, was identified as responsible for keratinocyte wound-healing stimulation [12]. Such wound-healing potential was also observed for ethanolic fruit pulp extract, once again scientifically validating one of the alleged properties of the plant in its ethnomedical uses [13].

The variety of bioactive phytochemicals with known antitumor efficacy in *C. guianensis* could anticipate the anticancer potential of its extracts. In reference to *Couroupita*, only one study has demonstrated the potential cytotoxic activity of its flower ethanolic and ethyl acetate extracts against MCF-7 breast cancer cell lines [14]. Isatin, an indole derivative isolated from the flowers of *C. guianensis*, has been shown to have strong antioxidant and anticancer activities against human promyelocytic leukemia (HL60) cells, inducing apoptosis [15].

Despite a decline in incidence and mortality rates in recent years, gastric cancer remains a significant health concern. The yearly impact of gastric cancer is projected to rise to 1.8 million new cases and approximately 1.3 million deaths by 2040. The prevalence of gastric cancer is notably higher in East Asia, particularly in China, where it accounts for a substantial proportion of cancer-related deaths [16]. The primary risk factor for gastric cancer is *Helicobacter pylori* infection, with dietary factors, smoking, obesity and genetic predispositions also contributing [17]. Despite advances in treatment, the prognosis remains poor, especially for late-stage diagnoses [18].

Conventional therapeutic modalities, including surgical intervention and chemotherapy, remain cornerstones in the management of gastric cancer, particularly in its early stages. Nevertheless, the advent of new molecular and immunological insights has paved the way for the development of promising innovative therapeutic strategies designed to target more advanced or metastatic gastric cancers. Immune checkpoint inhibitors like nivolumab and pembrolizumab or targeted therapies in specific patient populations, such as those focusing on HER2 and VEGFR2 signaling pathways, are now being used in combination with traditional chemotherapy to improve outcomes [18,19]. Prevention strategies focusing on reducing exposure to risk factors, implementing early screening programs, and finding new chemopreventive and therapeutic options are fundamental to mitigating the impact of gastric cancer.

Exploring and chemically characterizing traditional remedies, particularly those used in Amazonian shamanic medicine, could allow for pharmacological or adjuvant treatment of gastric cancer. Indeed, investigating traditional remedies integrates centuries-old medicinal knowledge with modern scientific research, which is useful for developing evidence-based herbal treatments. This approach can accelerate the discovery of effective treatments by leveraging ethnopharmacology.

Given these premises, in this study, we hypothesized that the aqueous bark extract of *C. guianensis* could have antitumor potential; therefore, we aimed to test its activity in a widely used in vitro model of gastric cancer, the AGS cell line, also focusing on the molecular mechanisms underlying its action, which have never been investigated to date. We found that the bark decoction of *C. guianensis* and the fraction III obtained by chromatographic fractionation, were highly effective in the inhibition of gastric cancer cells’ viability. The activity was dependent both on a direct inhibition of proliferation through cell cycle blockade and on the activation of apoptosis following autophagic flux engulfment.

## 2. Results

### 2.1. Production of Total Decoction (LDCG) of C. guianensis and Fractions and Evaluation of the Activity on Gastric Adenocarcinoma Cells’ Viability and Proliferation

The total decoction of *C. guianensis* (LDCG) was fractionated on a chromatographic column, using Sephadex LH-20 as the stationary phase. As reported in our previous work [12], fraction I (Fr I) corresponded to the pure molecule 4-(2″-O-sulfate- β-D-glucuronopyranosyl) ellagic acid, responsible for the effectiveness of the decoction in the keratinocytes’ wound-healing process. The fractionation procedure collected two further fractions (II and III). Based on retention time, UV/Vis data and a comparison of HRMS accurate masses and fragment ions’ MS/MS with standard compounds (when available) or literature data [20], the HPLC-UV-HRMS^n^ analysis suggested the occurrence of a gallic acid derivative (methyl brevifolin carboxylate) and 4-(2″-O-sulfate-β-D-glucuronopyranosyl) ellagic acid in Fr II and gallic acid and an ellagic acid glucoside sulfate derivative in Fr III (Supplementary Figure S1).

**Figure 1:**
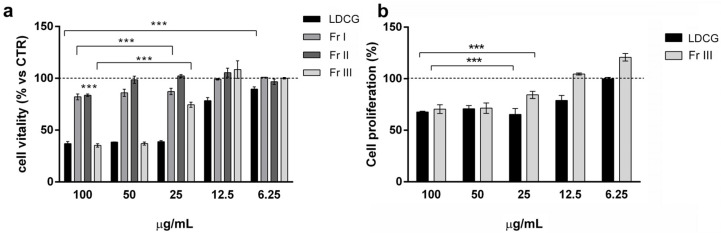
Evaluation of LDCG and fractions on AGS cellular viability and proliferation. Data were obtained by stimulating cells with different doses of LDCG and fractions (6.25–100 μg/mL) for 24 h, evaluated by MTT assay (**a**) and BrdU assay (**b**). Results are expressed as mean ± SD of 3 independent experiments performed in triplicate. One-way ANOVA was performed vs. control, followed by Tukey’s post hoc test (*** *p* < 0.001).

The viability assay on gastric adenocarcinoma (AGS) cells was performed to evaluate the toxicity of the decoction of *C. guaianensis* (LDCG) and its three major fractions in order to determine whether single molecules or the entire phytoextract is responsible for the observed effect. LDCG was able to significantly inhibit viability by up to 60% in the 25–100 μg/mL range. The IC50 of LDCG, calculated on the basis of the maximum observed effect, was 14.11 μg/mL (R^2^ = 0.97). As regards the fractions, only Fr III displayed an inhibitory activity comparable to the total LDCG, with an IC50 of 50.67 μg/mL (R^2^ = 0.95). In contrast, Fr I had a modest effect in the same dose range (about 20% inhibition), and Fr II was ineffective except at a 100 μg/mL dose (Figure 1).

Given that our previous study [12] demonstrated the presence of catechin derivatives (gallocatechin/epigallocatechin and methyl-epigallocatechin isomers), as well as ellagic and gallic acid, in LDCG, we conducted a viability assay treating cells under the same experimental conditions with these pure compounds at the indicated dose ranges. This was carried out to determine if the inhibitory activity of the LDCG could be attributed to these known compounds.

The data do not show significant activity for ellagic acid and catechin at all the doses tested. A slight reduction in cell viability, even if lower than that observed for LDCG and Fr III, was observed only with the highest dose of gallic acid, suggesting that the activity of LDCG and Fr III was not due to these compounds (Supplementary Figure S2) but can be attributed to the entire phytocomplex in which the set of active and inactive molecules may act synergistically and improve bioavailability.

We then assessed whether the reduction in cell viability was related to the direct inhibition of cell proliferation. AGS cells were cultured in the same concentration range and treatment time as for the MTT assay, and cell proliferation was assessed by BrdU incorporation.

Both LDCG and Fr III inhibited cell proliferation by 30–35% at the high doses tested (50–100 μg/mL), even though the effect was lower than that on cell viability (Figure 1b).

### 2.2. LDCG and Fraction III Directly Affect Cell Proliferation through Cell Cycle Blockade and Apoptosis Induction

**Figure 2:**
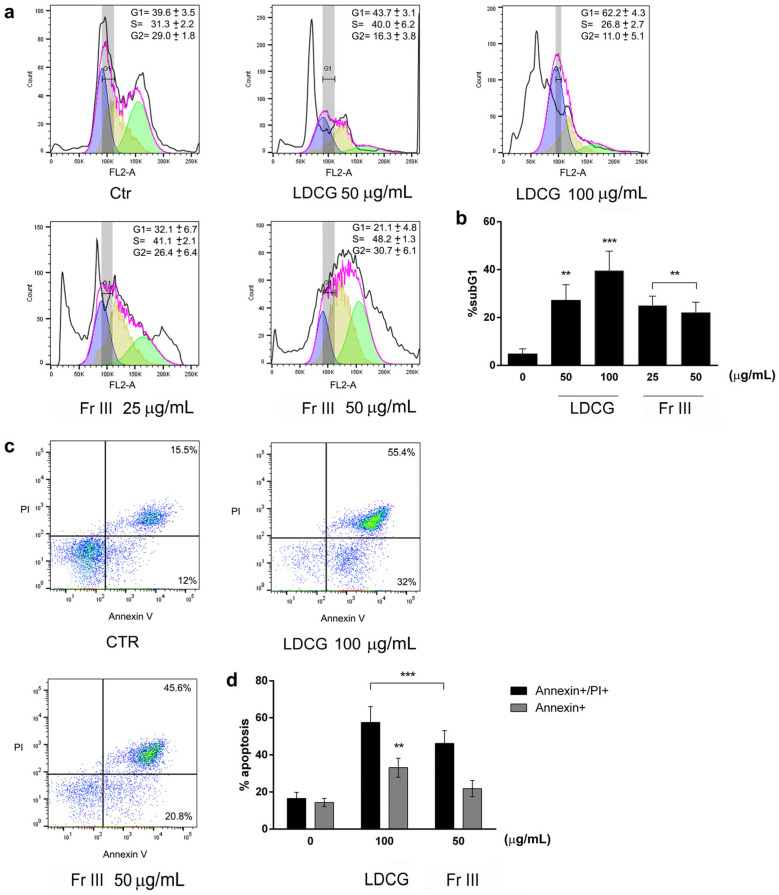
LDCG and Fr III induce a cell cycle blockade and apoptosis. (**a**) Representative images of cell cycle analysis. AGS cells were cultured for 24 h with LDCG (50–100 μg/mL) and Fr III (25–50 μg/mL). Flow cytometric analysis shows the percentage of cells in the G0/1, S and G2 phase of the cell cycle measured by FlowJo cell cycle analysis software through univariate modeling (magenta line). The mean percentage of the cells in each phase of the cell cycle ± SD (G1 = blue peak; S = yellow peak; G2 = green peak) is indicated each graph. (**b**) Bar graphs show the percentage of dead cells (sub-G1) determined by cell cycle analysis, expressed as mean ± SD of three independent experiments. (**c**) Representative images of annexin V and propidium iodide (PI) apoptosis analysis. The percentage of cells in apoptosis is reported on the dot plots. (**d**) The bar graph indicates the percentage of early (annexin V-positive cells/PI-negative cells) and late apoptotic events (annexin V/PI-double positive cells) vs. control cells. Data are expressed as mean ± SD of three independent experiments. One-way ANOVA was performed vs. control, followed by Tukey’s post hoc test (** *p* < 0.01, *** *p* < 0.001).

To understand how LDCG and Fr III inhibited cell viability and proliferation, we evaluated their effects on cell cycle progression and apoptosis by using flow cytometry assays (Figure 2). LDCG induced a G1-phase blockade at both concentrations tested, with a parallel increase in sub-G1 apoptotic cells. Fr III elicited an S-phase arrest, also increasing sub-G1 apoptotic cells, even if to a lesser extent (Figure 2a,b). The annexin/PI double-staining assay also confirmed apoptosis induction. Indeed, both LDCG and Fr III significantly increased the percentage of both early and late apoptotic cells (Figure 2c,d).

### 2.3. LDCG and Fr III Affect Key Cell Cycle Control Proteins and Promote the Expression of Apoptosis Machinery

To confirm the flow cytometry data on cell cycle arrest and apoptosis induction, we analyzed the expression of proteins specifically involved in these cell processes by Western blot.

**Figure 3:**
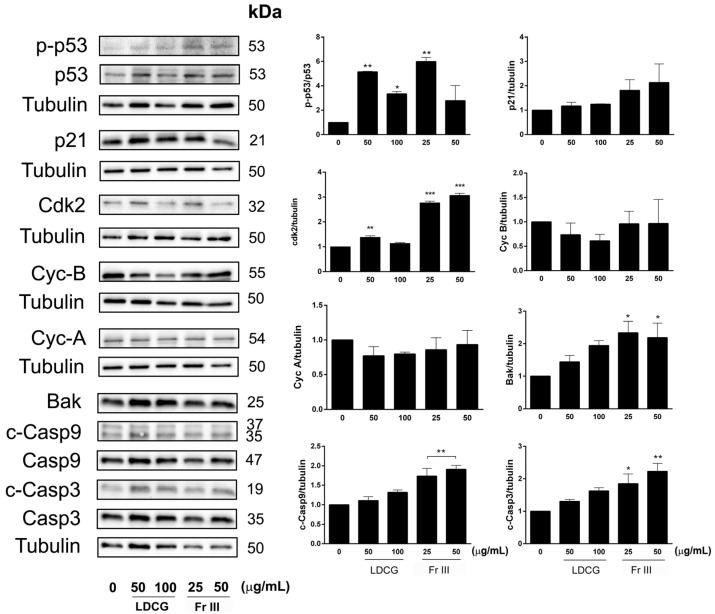
Expression of cell cycle and apoptosis proteins. AGS cells were treated with LDCG (50–100 µg/mL) and Fr III (25–50 µg/mL) for 24 h. Representative Western blot data of p-p53 (Ser15), p53, p21, CDK2, Cyc A, Cyc B, BAK, cleaved Casp9, Casp9, cleaved Casp3 and Casp3. Histograms represent the quantification of Western blot data. Tubulin was used as a loading control. Data were normalized to the values obtained for the control and were graphically represented as means ± SD of three independent experiments. One-way ANOVA, followed by Tukey’s post hoc test, was performed vs. control (* *p* < 0.05, ** *p* < 0.01, *** *p* < 0.001).

According to the proliferation assay and flow cytometry assay results, we observed a significant increase in the expression of the protein phospho-p53 after 24 h of treatment with both LDCG and Fr III and a parallel increased trend in its downstream target p21. Cdk-2 was significantly augmented by Fr III, which explains the S-phase cell cycle blockade. Cyclin B was significantly downregulated by LDCG, although Cyclin A showed only a slight inhibition, in agreement with the arrested progression of the cell cycle from the G1 phase. LDCG and Fr III dose-dependently induced the expression of critical pro-apoptotic proteins like Bak, cleaved caspase 9 and cleaved caspase 3. The effect reached statistical significance for the Fr III treatment. Therefore, the results show a different response among the treatments in terms of the expression of proteins involved in the cell death and cell cycle blockade processes (Figure 3).

### 2.4. LDCG and Fr III Promote the Autophagic Process

**Figure 4:**
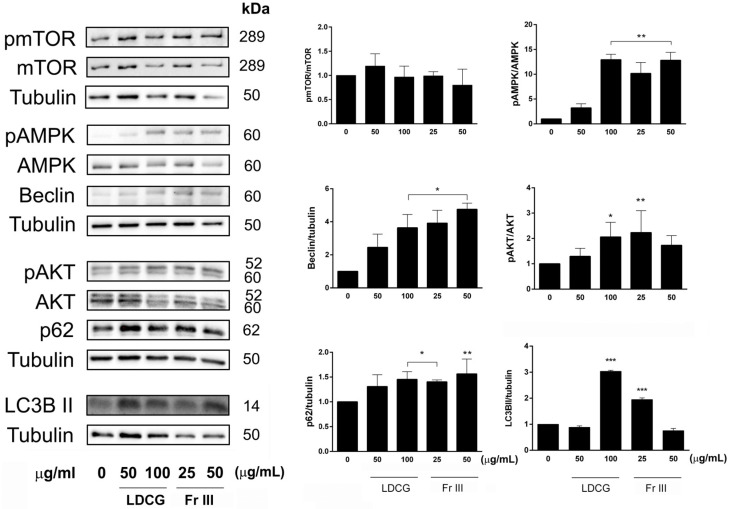
Expression of autophagic proteins. AGS cells were treated with LDCG (50–100 µg/mL) and Fr III (25–50 µg/mL) for 24 h. Representative Western blot data of p-AKT (Ser 473), AKT, p-mTOR (Ser 2448), mTOR, p-AMPK (Thr 172), AMPK, p62, Beclin and LC3BII are shown. Histograms represent the quantification of Western blot data. Tubulin was used as a loading control. Data were normalized to the values obtained for the control and were graphically reported as means ± SD of at least three independent experiments. One-way ANOVA, followed by Tukey’s post hoc test, was performed vs. control (* *p* < 0.05, ** *p* < 0.01, *** *p* < 0.001).

Given the activation of phospho-p53, a target protein that regulates the autophagic process beyond the apoptosis and arrest of cell cycle progression, we next analyzed its downstream target proteins involved in the autophagic flux [21]. We detected the activation of phospho-AMPK and phospho-AKT, converging in an upregulation of Beclin and LC3BII, the target proteins of autophagy induction, after treatment with LDCG and Fr III. mTOR expression was inhibited at the highest doses of both LDCG and Fr III, but its phosphorylation status and hence activation were not affected by treatment. Since LC3BII increment might be caused not only by autophagy induction but also by its engulfment at a later stage due to a hindrance in the turnover of autophagosomes, we analyzed the autophagic flux through the measurement of p62/SQSTM1 protein cargo expression, which is usually degraded in the later steps of autophagy [22]. We observed an accumulation of p62 following treatment with LDCG and Fr III, indicating a general blocking of the autophagic flux (Figure 4).

## 3. Discussion

*C. guianensis*, an evergreen tree whose various parts are used both in the shamanic practices of the “curanderos” of the Peruvian Amazonian and in ayurvedic medicine, through different formulations (decoctions, infusions, teas), has multiple potential therapeutic applications [1,2,3]. Its antibacterial, anti-inflammatory, and antioxidant properties have been backed by in vitro and in vivo studies in *C. guianensis* of Indian origin [4,23,24,25]. Few studies have been carried out on Amazonian *C. guianensis*, reporting antinociceptive, anti-inflammatory, and wound-healing properties [7,12,26].

Indeed, we recently reported an unprecedented phytochemical composition of the *C. guianensis* bark decoction as employed in Peruvian Amazonian traditional medicine, rich in polyphenolic compounds such as glycosides and sulfate derivatives of catechins and ellagitannins, identified for the first time in the genus of Lecythidaceae. Quantitative UHPLC-UV analysis revealed that the decoction contains ellagic acid derivatives, with concentrations ranging from 1.9 to 26.6 μg/mg, including sulfated forms at 203.8 ± 22.5 μg/mg. The total *C. guianensis* decoction and one of its main polyphenolic derivatives identified, namely 4-(2″-O-sulphate- β-D-glucuronopyranosyl) ellagic acid, were found to be particularly promising in promoting skin wound healing [12].

In the present study, we aimed to substantiate the alleged antitumor efficacy of the bark decoction and its derived fractions I-III in the treatment of gastric cancer, which have never been investigated to date since previous studies only reported the cytotoxic activity of organic flower extracts in other tumor models without proposing a mechanism of action [16,17]. In general, the specific compounds in *C. guianensis* that contribute to its anticancer effects include a variety of bioactive phytochemicals, from flavonoids and phenolic compounds to saponins and alkaloids. In particular, isatin, an indole derivative extracted from *C. guianensis* flowers, has been reported to induce apoptosis in human promyelocytic leukemia (HL60) cells [15]. Quercetin and stigmasterol isolated from the methanol flower extract were reported to be potentially responsible for the cytotoxic effect observed in several cancer cell lines like Hela and HepG2 [27]. Ethanolic-ethyl acetate flower extracts were cytotoxic against MCF-7 breast cancer cells [14]. Even if these few studies substantiated the anticancer potential of *C. guianensis*, they were performed only on flower organic extracts, and no mechanism of action was provided.

Here, we reported for the first time the anticancer efficacy of the bark decoction (LDCG), as prepared in shamanic traditional medicine, and provided a mechanism for the cytotoxic activity observed, which implied antiproliferative, pro-apoptotic, autophagic and cell cycle blockade effects. It is noteworthy that the effect on cell viability by the total decoction was not dependent on the activity of its best-known compounds (ellagic acid, gallic acid and catechin). Natural polyphenols, such as ellagic acid derived from pomegranate, have demonstrated antitumor properties both in vitro and in vivo against gastric cancer cells. These effects include the induction of apoptosis, the inhibition of cell migration, the regulation of gene expression associated with programmed cell death and the suppression of inflammatory gene expression [28]. Previous reports have indicated that gallic acid can inhibit the growth of various cancer cells, including prostate, cervical and colon cancer, as well as melanoma and leukemia cells [29]. The molecule induced apoptosis in AGS cells, mediated by the upregulation of Fas, FasL and DR5 expression, the activation of members of the caspase family (caspase 3 and caspase 9), and the downregulation of small mitochondrial proteins (Bad, Bak, cyt c) [30]. In our study, however, it was observed that gallic acid only exerts activity at the highest dose, while ellagic acid and catechin did not show significant activity at any of the doses tested.

Fr III, composed of gallic acid and an ellagic acid glucoside sulfate, showed similar but lower cytotoxic activity than LDCG. These observations confirm that inhibitory activity depends more on the action of the set of active and inactive molecules in the decoction, the so-called phytocomplex (of which Fr III is only a part), which is able to enhance molecular synergism and improve the bioavailability of LDCG. Moreover, the full characterization of total LDCG using advanced chromatographic techniques such as HPLC-UV-HRMS^n^ allowed us to establish the phytocomplex fingerprint and identify the main compounds that may contribute to the overall bioactivity of the medicinal plants.

Ellagic acid glycosylated sulfate derivatives are relatively rare. They are found exclusively in genera belonging to a few specific botanical families, namely Rosaceae, Tamaricaceae, Frankeniaceae, Lythraceae, Combretaceae and Myrtaceae [31,32]. In general, ellagic acid glycosylated derivatives have been synthetically produced to enhance the bioactivity and solubility of ellagic acid, which limits its pharmaceutical and cosmetic applications. A study on the synthesis of ellagic acid glucoside via transglycosylation using sucrose and glucansucrase demonstrated its neuroprotective potential, with enhanced water solubility and stronger effects against acetylcholinesterase compared to ellagic acid itself [33]. Okicamelliaside is a natural antiallergic ellagic glycoside isolated from the leaves of *Camellia japonica* that displays antidegranulation activity and was recently reported to be a natural inhibitor of HSP90 with antitumor activity due to the selective inhibition of the HSP90–CDC37 protein complex [34,35].

The reduction in cell viability by LDCG and its active Fr III could be linked not only to reduced cell proliferation but was also to the activation of the apoptotic process and the blocking of cell cycle progression, as confirmed by the increased expression of proteins linked to these processes, like p-p53, cdk2, p21, Bak and the activated caspases. Even if Fr III is capable of exerting anticancer action on its own, total LDCG has a cytotoxic and pro-apoptotic activity greater than that of Fr III at comparable doses, confirming the importance of the entire phytocomplex compared to single or mixtures of isolated bioactive molecules.

Interestingly, total LDCG and the active fraction III both inhibited proliferation and induced the apoptotic process, yet they blocked the cell cycle at different stages. Indeed, LDCG inhibited the progression of the cells from the G1 phase, whereas Fr III induced an S-phase arrest.

Although cell cycle, autophagy and apoptosis represent distinct cellular processes with important biochemical and morphological differences, the key proteins that control their regulation and execution can interact to form a network. The interplay between the processes and the final outcome depends on the interaction of proteins that influence these processes to try to maintain cellular homeostasis or in response to cell stress, like that caused by pharmacological treatments. Cancer cells, which often rely on autophagy to cope with their high metabolic demands and stressful tumor microenvironment, are particularly vulnerable to disruptions in this process. AMPK typically promotes autophagy by inhibiting mTOR, which is a known suppressor of autophagy. We found that mTOR was not affected by treatment with either the decoction or Fr III. This could be due to the fact that in AGS cells, mTOR is overexpressed due to the upstream overexpression of PIK3CA and AKT1 or loss of PTEN, which are altered in this cell line [36]. In the scenario where pAMPK is activated but pmTOR is not inhibited, this suggests a paradoxical signaling event where the usual inhibitory effect of pAMPK on mTOR is bypassed or overridden. In this context, autophagy initiation might still occur due to pAMPK activation, but the residual pmTOR levels likely hinder the later stages of autophagy, leading to a blockage of autophagic flux with a potential metabolic stress response in tumor cells that triggers apoptosis [37].

Therefore, the inhibition of the autophagic flux that we observed upon LDCG and Fr III treatment can selectively induce apoptosis, contributing to the anticancer effect. Several studies have shown that natural products can inhibit carcinogenesis by regulating autophagy. For instance, a recent study found that the flavonoid apigetrin induces autophagic cell death in AGS human gastric cancer cells through the PI3K/AKT/mTOR pathway [38]. Polysaccharides from *Ganoderma lucidum* were reported to display an anticancer effect in cancer due to the inhibition of autophagic flux through LC3II and p62 accumulation [39]. Therapies, combining autophagic flux inhibition with apoptosis induction, do indeed offer promising avenues for effective gastric cancer treatment.

Further studies, including in vivo ones, will be necessary to discover the full anticancer potential of *C. guianensis* in gastric cancer and to explore in deeper detail the signaling mechanisms underlying its biological effects.

The exploration and chemical characterization of plants employed in traditional remedies, particularly in shamanic Amazonian medicine, have been reported as valuable for the treatment of several diseases [40,41]. These traditional remedies are often derived from diverse plant species with unique bioactive compounds that have evolved to combat various pathogens and diseases. The Amazonian rainforest is a vast reservoir of biodiversity, harboring numerous plant species with potential medicinal properties. Historically, shamanic practices have utilized these plants for their therapeutic benefits, which modern science is now beginning to explore in depth. One key relevant feature of these traditional remedies is their potential to offer novel chemical entities that could be developed into anticancer agents. These natural compounds may have fewer side effects than synthetic drugs, offering a more holistic approach to treatment.

Furthermore, understanding the mechanisms of action of these traditional remedies can provide insights into new therapeutic targets and pathways at the molecular level, potentially leading to the development of more effective treatments.

## 4. Materials and Methods

### 4.1. Plant Material, Decoction Preparation and HPLC-UV-HRMS^n^ Analysis

The bark of *C. guaianensis* was collected from the primary forest of Mayantuyacu in the Ucayali Region, Peru, in October 2019 and identified by Prof. Teresa Mencherini. A voucher specimen (CG_B_2022) was stored at the University of Salerno, Department of Pharmacy. The preparation of the decoction was carried out through the standardized procedure, as reported by T. Esposito et al. [12]. Briefly, 50 g of bark was cut and added to 1 L of boiling water, steeped for 10 min, then filtered and freeze-dried (lyophilizer Alpha 1–2 LD freeze dryer, Martin Christ, Osterode am Harz, Germany). The preparation yield was 5.2% (*w*/*w*) of the initial bark material. The fractionation of the decoction on Sephadex LH-20 using water as eluent, leading to the main fractions I-II and III, and their qualitative and quantitative analysis through HPLC-UV-HRMS^n^ techniques were conducted under the previously reported experimental conditions [12].

### 4.2. Cell Culture and Treatments

Gastric adenocarcinoma (AGS) cells (ATCC CRL-1739) were obtained from ATCC (Manassas, VA, USA). Cells were cultured in RPMI medium (Microgem, Naples, Italy) with 10% (*v*/*v*) heat-inactivated fetal bovine serum (Microgem), 50 U/mL penicillin and 50 μg/mL streptomycin (Microgem) and 2mM L-glutamine (Gibco, Grand Island, NY, USA). Cells were maintained at 37 °C in a 5% CO2 and 95% air-humidified atmosphere and passed twice a week. Cells were seeded at a density of 1.4 × 10^4^/cm^2^ to perform treatments and cultured for 24 h before stimulation. LDCG and fractions were used in a range of doses (0.8–100 μg/mL) to assess their cytotoxic effect. A control group of cells (control group) were stimulated with medium alone, and the experiments were conducted over 24 h of exposure. Ellagic acid, gallic acid and catechin standards were purchased from Sigma-Aldrich (Merck KGaA, Darmstadt, Germany).

### 4.3. Cell Viability Assay

Cytotoxicity was assessed by performing the 3-(4,5-dime-thylthiazol-2-yl)-2,5-diphenylte-trazolium (MTT) assay (Sigma-Aldrich, St. Louis, MO, USA) [42]. AGS cells (5 × 10^3^/well) were cultured at 37 °C for 24 h in 96-well plates before adding treatments. After 22 h of treatment, 0.5 mg/mL of MTT was added to the cell medium, and plates were incubated for the last two h at 37 °C and 5% CO2. The resulting formazan crystals were dissolved in 100 μL/well of solubilization solution (10% Triton X-100 and 0.1 N HCl in isopropanol). The absorbance of the resulting cell suspension was measured at 570 and 650 nm to subtract the background in each sample on a TECAN Infinite M200Pro microplate reader (Tecan Trading AG, Männedorf, Switzerland). Cell viability was assessed in triplicate and expressed as a percentage versus the untreated control cells (100%).

### 4.4. BrdU Assay for Cell Proliferation Determination

AGS cells (5 × 10^3^/well) were cultured at 37 °C for 24 h in 96-well plates before adding treatments. A BrdU assay (BrdU colorimetric assay kit; Roche Applied Science, South San Francisco, CA, USA) was used to measure cell proliferation, determined by an ELISA (enzyme-linked immunosorbent assay) on a TECAN Infinite M200Pro microplate reader at 370 nm (reference 492 nm). Cell proliferation was assessed in triplicate and expressed as a percentage versus the untreated control cells (100%).

### 4.5. Flow Cytometry Assays

For the quantitative assessment of apoptosis, cell death and cell cycle progression, the AGS cells were cultured in p60 tissue culture plates for 24 h in RPMI 10% fetal bovine serum at 37 °C. To identify apoptosis, cells were stained by antihuman annexin V-FITC (Dojindo Laboratories, Tokyo, Japan) and propidium iodide (PI) (Sigma-Aldrich, St. Louis, MO, USA). After treatment, cells were harvested with PBS-EDTA, washed twice with PBS, and resuspended with annexin V buffer, annexin V-FITC, and PI for 15 min before flow cytometric analysis on BD FACSVerse (BD Bioscience, Franklin Lakes NJ, USA) [43]. Cells were collected as described above, washed twice with PBS, and resuspended in PBS-EtOH (70%) for 24 h at −20 °C to analyze cell cycle progression. Later, cells were stained with PI for 15 min before flow cytometric analysis. The results were analyzed with FlowJo software v. 10 (BD Bioscience). The cell cycle Watson Pragmatic algorithm was used to measure cell cycle phase distribution [44].

### 4.6. Western Blot

As described previously, the cell medium was removed at the end of the treatment, and adherent cells were washed with PBS [45]. After washing, cells were lysed in ice-RIPA buffer solution (Bio Rad, Hercules, CA, USA) supplemented with both protease and phosphatase inhibitors (Sigma-Aldrich). Cells were also lysed mechanically with a scraper in ice, collected in a centrifuge tube, and later lysed with an insulin syringe to help break down cells. Cells were centrifuged to remove debris, and the total protein extract was estimated using the Bio-Rad Protein Assay. The antibodies used for analyses were α-Tubulin (1:5000, Sigma-Aldrich); phospho-p53 (p-p53 Ser15, #9286cs, Cell Signaling Technologies, MA, USA), p53 (#2527cs), p21 (#2947cs), cdk2 (#2546cs), CycB (#4135cs), CycA (#596cs), BAK (#6947cs), Casp9/c-Casp9 (1:500, #9502cs), Casp3 (#9662cs), c-Casp3 (#9661cs), phospho-Akt (p-Akt; Ser473, #4060cs), AKT (#4691cs), phospho-mTOR (p-mTOR Ser 2448, #5536cs), mTOR (#2983) phospho-AMPK (p-AMPK, Thr172, #2535cs), AMPK (#2603cs), Beclin 1 (#3495cs), p62 (#P0067 Sigma-Aldrich) and LC3BII (1:500, #2775cs). Unless otherwise indicated in brackets, all antibodies were used at 1:1000 dilution. Secondary HRP-conjugated antibody goat anti-mouse or goat anti-rabbit IgG was obtained from Bio-rad. The membranes were then stained using a chemiluminescence system (Amersham ECL, Cytiva, MA, USA) and acquired with the Chemidoc MP System (Bio-rad).

### 4.7. Statistical Analysis

Statistical analyses were performed by using GraphPad Prism 6.0 software. Data analyses were obtained as mean ± SD and analyzed with one-way ANOVA followed by Tukey’s post hoc analysis. Data were considered statistically significant when the *p* value was inferior to 0.05 (* *p* < 0.05).

## 5. Conclusions

In conclusion, the data reported in this work showed evidence of new biological activities and mechanisms of action related to the bark decoction of *C. guianensis* (‘ayauma’), which is traditionally employed in shamanic Amazonian medicine, in an in vitro model of gastric adenocarcinoma.

Discovering new pharmacological remedies from natural sources for gastric cancer is a matter of urgency due to its high impact, and developing evidence-based herbal remedies appears essential for expanding innovative treatments, overcoming drug resistance, reducing side effects and leveraging the vast chemical diversity of natural compounds. This endeavor holds promise for improving patient outcomes and underscores the value of integrating traditional millenary knowledge with cutting-edge scientific research.

## Data Availability

Data are available on request.

## Supplementary Materials

The following supporting information can be downloaded at https://www.mdpi.com/article/10.3390/ijms25179183/s1.
