# Supplementary material for: Anticancer Activity and Mechanism of Action of Couroupita guianensis Bark Decoction in Gastric Adenocarcinoma Cancer Cell Line
Source: Int J Mol Sci. 2024 Aug 24;25(17):9183. doi: 10.3390/ijms25179183 (PMC11395395; doi:10.3390/ijms25179183)
Supplement: Supplementary file 1 [file ijms-25-09183-s001.zip › ijms-3164769-supplementary.pdf]

Supplementary Figures

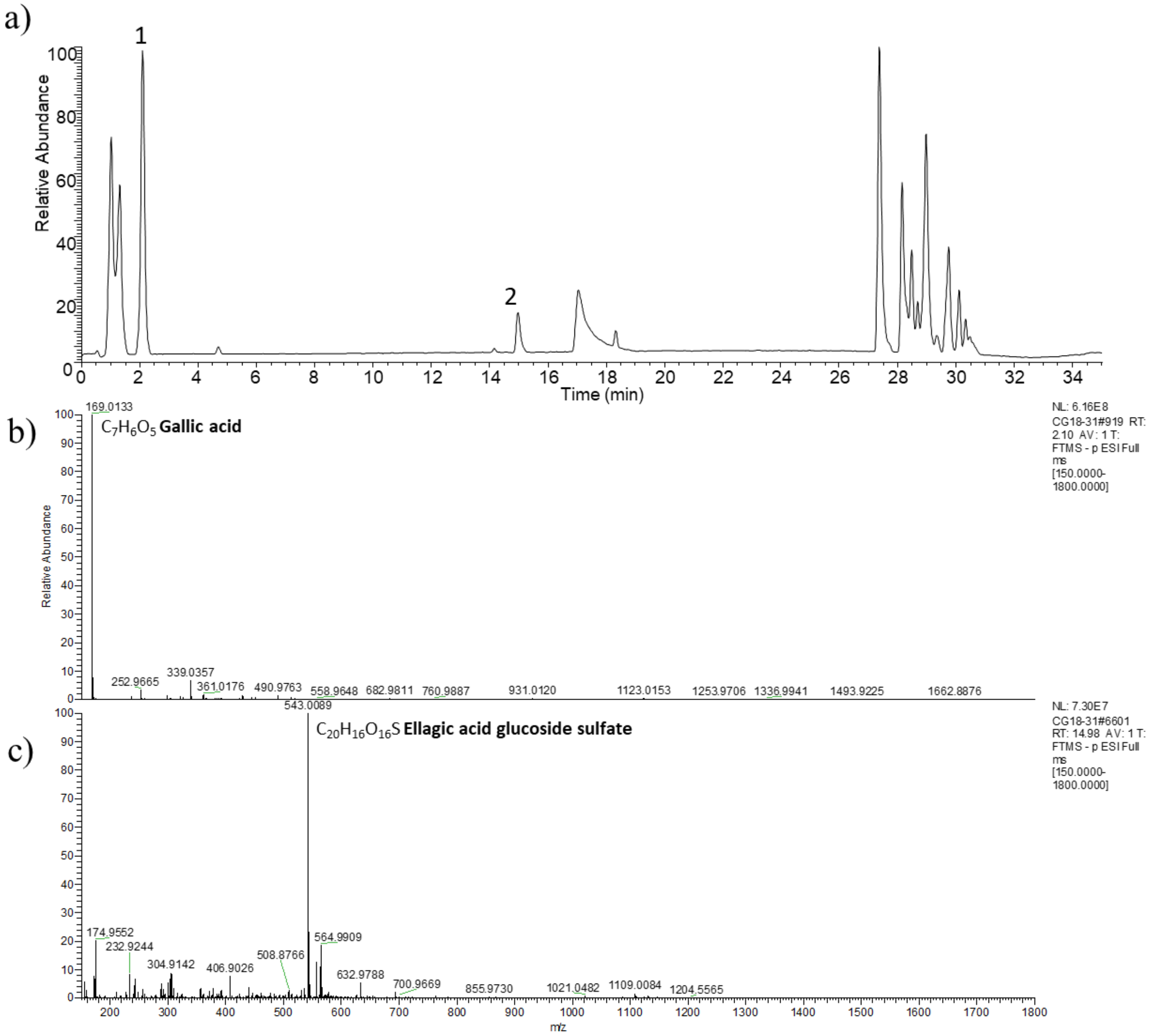

Supplementary Figure S1. a) UHPLC-HRMS profile of Fraction III, b and c) MS spectra of peak 1 (gallic acid) and peak 2 (ellagic acid glucoside sulfate).

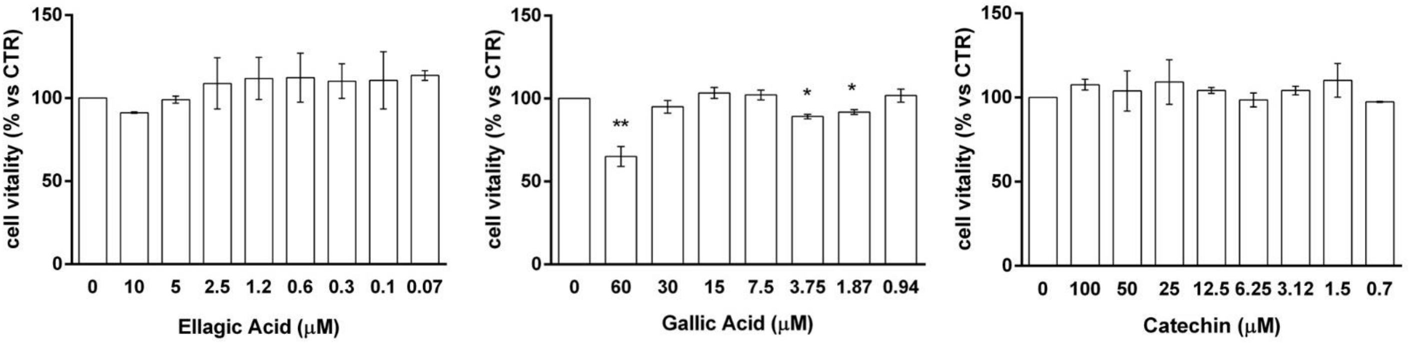

Supplementary Figure S2 Evaluation of gallic acid (0.94-60 μM), ellagic acid (0.07-10 μM), catechin (0.7-100 μM) for 24 h, evaluated by MTT assay. Results are expressed as media ± SD of 3 independent experiments performed in triplicate. One-way ANOVA analysis was performed *vs.* control followed by Tukey's post-test (*p<0.05, **p<0.01, ***p<0.001).
